# Co-designed PICU Family Stress Screening and Response System to Improve Experience, Quality, and Safety

**DOI:** 10.1097/pq9.0000000000000145

**Published:** 2019-03-07

**Authors:** K. Ron-Li Liaw, Jeanne Cho, Lea Devins, Jennifer Daly, Dennis Sklenar, Yasir Al-Qaqaa

**Affiliations:** From the * Sala Institute for Child And Family Centered Care, NYU Langone Health, New York, N.Y.; † Department of Child and Adolescent Psychiatry, NYU Langone Health, New York, N.Y.; ‡ Department of Nursing, NYU Langone Health, New York, N.Y.; § Department of Social Work, NYU Langone Health, New York, N.Y.; ¶ Department of Pediatrics, NYU School of Medicine, New York, N.Y.

## Abstract

**Objective:**

Evidence for successful and sustainable models that systematically identify and address family stress in the pediatric intensive care unit (PICU) remains scarce. Using an integrated improvement science and family engagement framework, we implemented a standardized family stress screening tool and response protocol to improve family experience and reduce family crises through the timely coordination of parent support interventions.

**Methods:**

We conducted this improvement initiative in the 12-bed PICU of a children’s hospital within a large, urban academic medical center. Our team, which included 2 family advisors, adapted a validated Distress Thermometer for use in pediatric intensive care. A co-designed family stress screening tool and response protocol were iteratively tested, refined, and implemented in 2015–2017. Process and outcome measures included screening and response reliability, parent satisfaction, and security calls for distressed families.

**Results:**

Over the 18 months, the percentage of families screened for stress increased from 0% to 100%. Among families who rated stress levels ≥5, 100% received the recommended response protocol, including family support referrals made and completed within 24 hours of an elevated stress rating. From 2015 to 2017, PICU parent satisfaction scores regarding emotional support increased from a mean score of 81.7–87.0 (*P* < 0.01; 95% CI). The number of security calls for distressed families decreased by 50%.

**Conclusions:**

The successful implementation of a co-designed family stress screening tool and response protocol led to the timely coordination of parent support interventions, the improved family perception of emotional support, and reduced family crises in the PICU.

## INTRODUCTION

Over the past 4 decades, medical and technological advances have significantly improved survival rates in pediatric critical care; however, a growing body of evidence demonstrates the wide-ranging, long-term psychologic effects on the child and family following pediatric intensive care unit (PICU) admissions. Parents of PICU patients have increased risks of posttraumatic stress disorder (PTSD), anxiety, and depression, which can lead to an inability to work, loss of earnings, and chronic ill health.^1–3^

In a review of PTSD in children and their parents following PICU admission, parental PTSD prevalence rates ranged between 10% and 21%, with subclinical symptom rates approaching 84%.^2,4–8^ Nelson and Gold^2^ posit that “the PICU environment likely creates a dynamic series of traumatizing events that influence both the child and the family” given the life-threatening nature of the illness and complex diagnostic workups and treatments, which often include daily invasive interventions.^2^

There are also well-established linkages between parental and child mental health.^4,9–13^ The subjective level of distress experienced by parents during PICU admission has been identified as an important and potentially modifiable risk factor in the development of parental mental health disorders, subsequent functional impairment, and importantly, also poor child emotional and behavioral outcomes.^4,11,14–18^ Implementing a universal screening tool that identifies parents with high levels of distress and psychosocial risk during the acute period of illness or injury provides a unique opportunity for early intervention and timely linkage to psychosocial resources and ongoing support.^19^ Evidence for successful and sustainable models that systematically identify and address parental distress during pediatric intensive care remains scarce and of critical need.^20^

In 2013, following a series of adverse events involving distressed families in the PICU, a quality research team conducted a gap analysis to identify PICU clinician, staff, and family caregiver perspectives. Several themes emerged from the gap analysis, including inconsistency in the approach to screening for families’ degree of stress/coping, insufficient integration of psychosocial and medical aspects of care, unclear escalation protocols for managing family crises, and a nonstandardized approach to addressing family stressors both inside and outside of the hospital. Given a lack of established national best practices and locally identified opportunities for improvement, an interdisciplinary quality improvement (QI) team at the Hassenfeld Children’s Hospital at NYU Langone, which included family advisors, aimed to test, implement, and sustain the use of a co-designed family stress screening and response system.

### Objectives

Our primary aim was to increase the percentage of families screened for stress during PICU stays, using a standardized family stress screening tool, from a baseline rate of 0%–90% during the 18-month QI initiative. A secondary aim was to increase the percentage of families who received recommended interventions based on a standardized response protocol, triggered by a stress score of ≥5, from a baseline rate of 0%–90%.

## METHODS AND CONCEPTUAL FRAMEWORK

We used the Institute for Healthcare Improvement’s Model for Improvement as the guiding framework and incorporated learning from iterative Plan-Do-Study-Act (PDSA) cycles to test and refine interventions.

### Setting and Context

We conducted the QI initiative in the 12-bed medical and surgical PICUs of a children’s hospital within a large, urban academic medical center in New York City. Patients from the Congenital Cardiovascular Care Unit were not included in this phase of the initiative. From 2013 to 2018, the children’s hospital made strategic investments to enhance psychosocial support and consultative services in pediatric social work, child life/creative arts therapy (CAT), integrative medicine, psychology, psychiatry, palliative care, and spiritual care.

### Improvement Team

In the fall of 2015, a PICU QI team was assembled consisting of PICU nurse leaders and champions, medical director, social worker, child life/creative art therapist, integrative health specialist, chaplain, psychologist, project manager, and 2 family advisors. The family advisors are parents with PICU hospitalization experience, who are employed and trained members of the hospital team. The team was led by a child psychiatrist trained in improvement science. Senior hospital leaders provided oversight, monitored progress, and helped to address systems-level barriers.

### Planning the Intervention

The QI team reviewed the literature on hospitalization and parental stress,^21–24^ focusing on validated screening tools and interventions for use within the PICU context. In 1997, the National Comprehensive Cancer Network developed the Distress Thermometer, now used widely within oncology care. This tool consists of a global screener of distress, an accompanying problem list, and treatment recommendations for psychosocial issues.^23,24^ Using a visual analog, self-report scale from 0 (no distress) to 10 (extreme distress), oncology patients are asked to circle the number that best indicates distress levels in the past week.^24^ Previously, a cutoff score of 5 was used to identify patients experiencing significant distress.^23^ Patel et al^25^ validated the use of an adapted pediatric Distress Rating Scale (DRS) against standardized measures within a pediatric oncology setting and established a tri-level distress classification of *mild* (DRS, 0–4), *moderate* (DRS, 5–7), and *severe* distress (DRS, 8–10). Haverman et al^26^ validated a Distress Thermometer for Parents of chronically ill children and determined a cutoff score of 4 for clinical distress in outpatient settings.

**Fig. 1.**
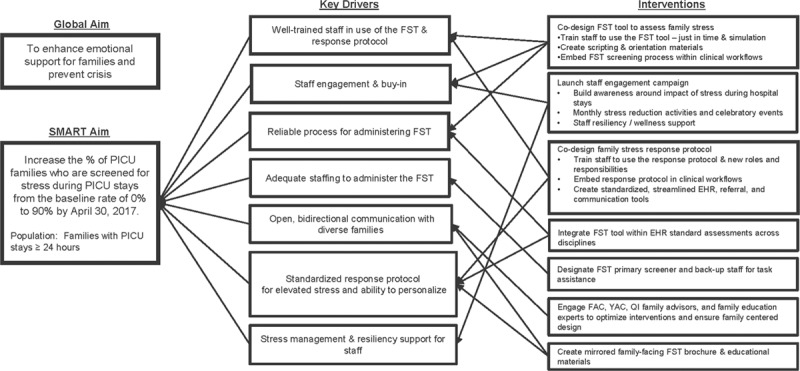
KDD to enhance emotional support for families and to prevent a crisis. KDD indicates key driver diagram. SMART; A SMART aim is an aim that is specific, measurable, achievable, relevant and timely. YAC; Youth Advisory Council.

Building upon the evidence of these established distress screening tools, the interdisciplinary QI team in collaboration with the hospital’s Family and Youth Advisory Councils co-designed a Family Stress Thermometer (FST) and accompanying response protocol for elevated scores. The FST was designed with parents of PICU patients as the target screening audience but also to be inclusive of other family members and non-family caregivers present at the bedside. Important co-designed FST adaptations included the use of the term *stress* rather than *distress* for broader acceptability, removal and addition of common stressors based on relevance within an intensive care setting, and transition from a self-report tool to a semi-structured, staff-facilitated conversation. The interdisciplinary QI team developed a key driver diagram to identify and prioritize interventions needed to achieve our specific aims (Fig. 1). Key drivers identified included having an engaged PICU and psychosocial staff well-trained in the use of the FST and response protocol, adequate staffing and reliable process for FST administration, a standardized response protocol that can be personalized for each family’s unique needs, open and bidirectional communication with a diverse range of families, and stress management and resiliency support for staff.

### Interventions

**Fig. 2.**
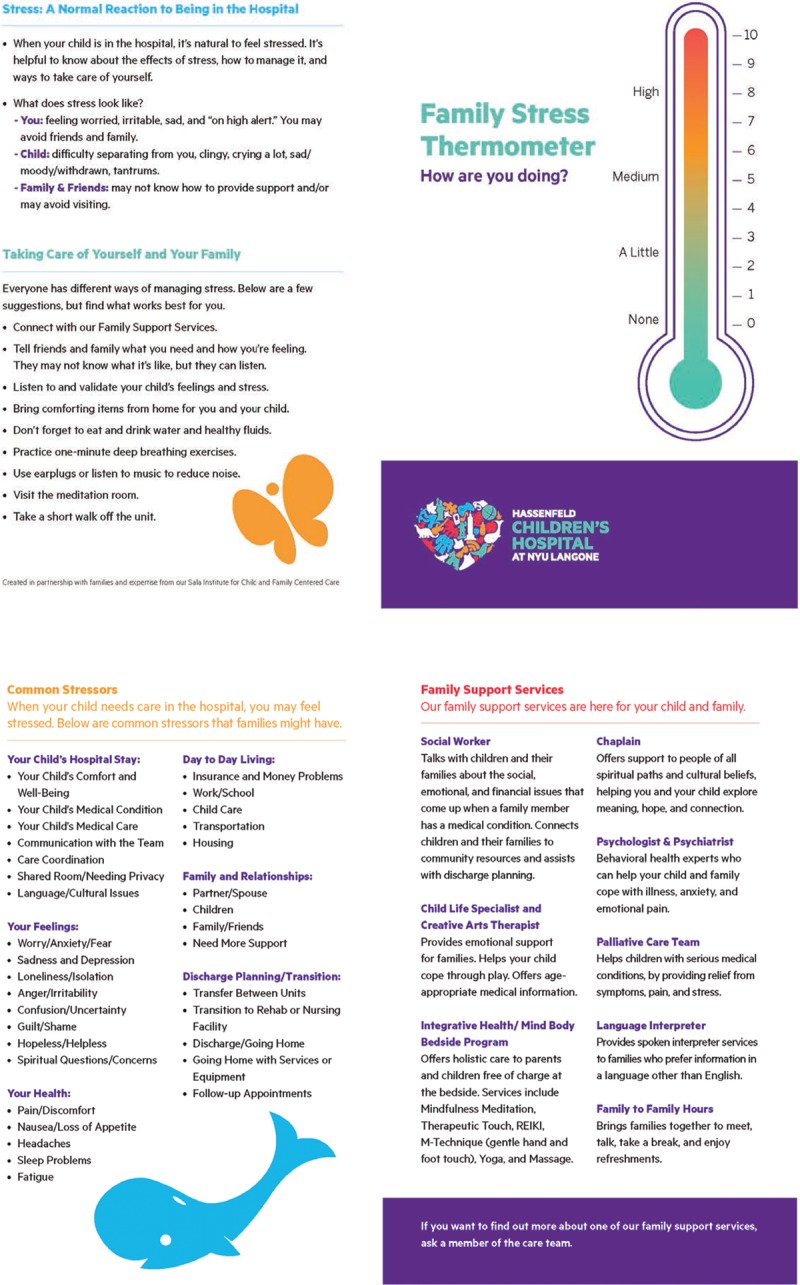
Family Stress Thermometer screening tool.

We developed the FST stress screening tool (Fig. 2) and response protocol through an evidence-based, iterative process, co-designing with family advisors and interdisciplinary staff, and testing and refinement through PDSA cycles. Key interventions included the following: (1) co-designed FST screening tool and response protocol; (2) staff scripting, education, and simulated case scenarios; (3) staffing availability for screening; (4) streamlined team communication and support service referral process; (5) family support resources and psychoeducational materials; and (6) electronic health record (EHR) integration. Other interventions not reported on within this report focused on stress management and resiliency for staff.

### Measures

#### Process Measure.

The primary process measure was the percentage of PICU families screened for stress during their PICU stay. Families with PICU stays <24 hours were considered “observation status” and therefore not included in the new screening process. A second process measure was the percentage of families with stress scores ≥5, who received recommended interventions based on a standardized yet personalized family stress response protocol. Additional analysis included the percentage of families referred to and seen by psychosocial support services within 24 hours of reporting an elevated stress score.

#### Outcome Measures.

The primary outcome measure was parent-reported satisfaction scores for the “degree to which staff addressed your emotional needs.” We extracted the satisfaction scores from the Press Ganey database. A secondary outcome measure was the annual number of security calls for distressed families and visitors.

### Analysis

Statistical process control was utilized to track the primary process measure of the percentage of families screened for stress during a PICU admission displayed on a P-chart. Established rules were used to differentiate special versus common cause variation. A chart review was conducted to assess compliance with the recommended response protocol for families with stress scores ≥5 during June and July of 2016 following unit-wide implementation. For our primary and secondary outcomes, we analyzed the absolute change in percentage from baseline (2015) to 2017.

## RESULTS

### Co-designed Family Stress Screening Tool

#### FST Version 1.

**Fig. 3.**
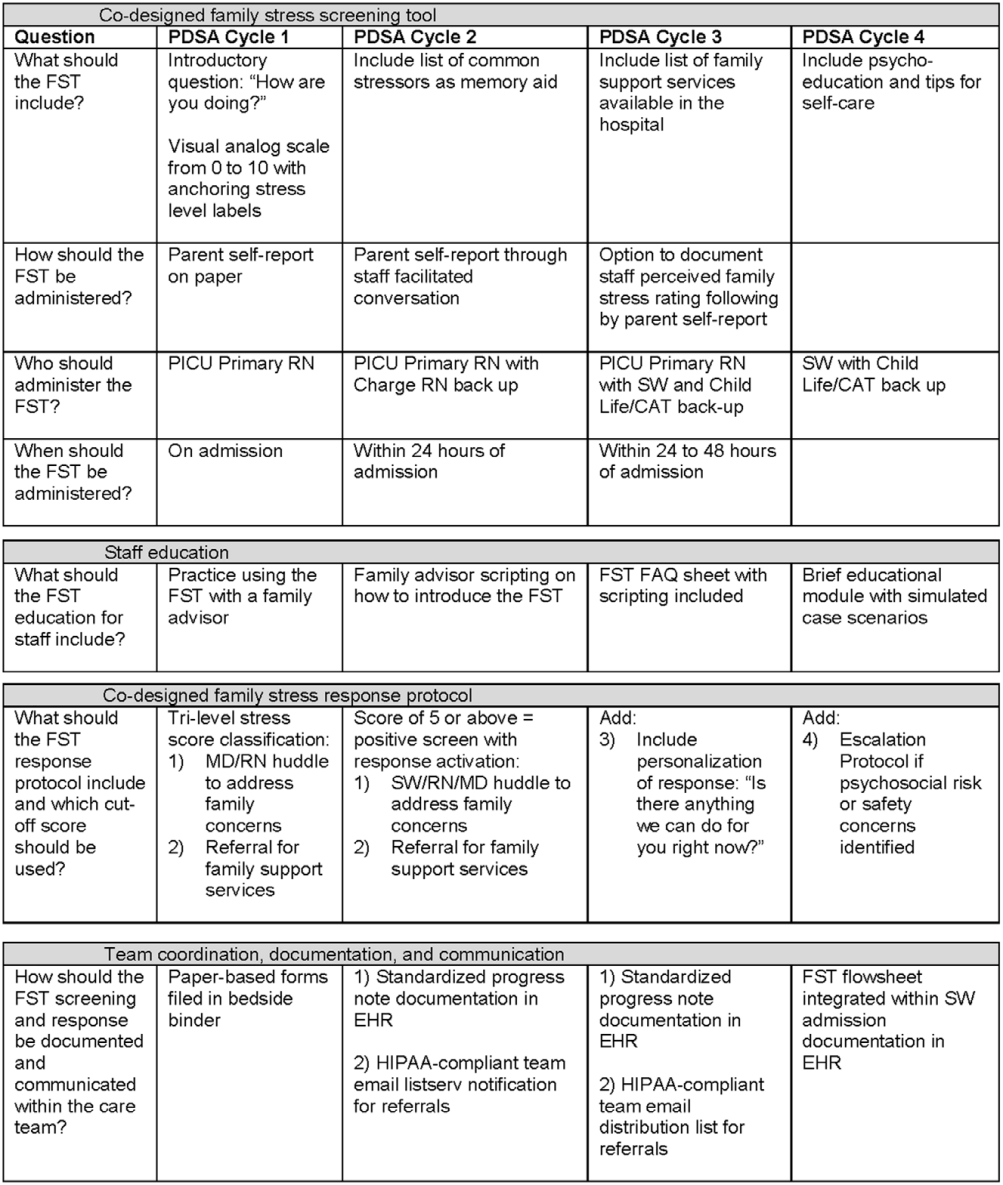
PDSA cycle ramps completed to test and optimize key elements of family stress screening and response. FAQ indicates frequently asked questions; HIPAA, Health Insurance Portability and Accountability Act; MD, medical doctor; RN, registered nurse; SW, social work.

The tool began with the introductory question, “How are you doing?” and asked families to rate their level of stress over the past 24 hours on a 0–10 scale (no to high stress). Similar to the National Comprehensive Cancer Network Distress Thermometer, this early version included a list of common stressors with checkboxes. It was administered as a self-report tool on paper with a brief introduction provided by PICU staff. Barriers and challenges encountered while testing the intervention included the length of time (15–30 minutes) it took to explain the instructions and complete the screen, particularly for families with high-stress ratings. PICU parents also gave feedback that the screening “felt like homework” during an already stressful time (Fig. 3).

#### FST Version 2.

We gathered parent self-reported stress levels through a semi-structured, staff-facilitated conversation introducing the FST tool and list of common stressors with checkboxes removed (Fig. 3). Families reported that having a “memory jogger” list of common stressors helped them better identify and articulate specific challenges that their families were facing.

In subsequent PDSA cycles, the FST incorporated a list of psychosocial support services within the hospital, basic psychoeducation on the impact of stress during hospitalization, and tips for self-care. Upon further testing in high-stress situations, the QI team created an option for staff to document a *perceived stress score* on behalf of families followed by a parent self-report score in subsequent encounters.

The FST tool and process were developed throughout multiple PDSA cycles refining specific aspects of the implementation such as who should conduct the family stress screen?; at what point during the PICU stay should families be screened?; and how best should we roll out education on this new screening tool and process? (Fig. 3).

We identified multiple barriers and challenges when the PICU nurses were tasked with conducting the family stress screening, such as understaffing, competing clinical and safety priorities, and decreased clinical efficiency. Subsequent PDSA cycles identified the pediatric social worker as the most reliable family stress screener given their expertise in assessing family stress/coping and ability to address family needs.

### Co-designed Family Stress Response Protocol

#### Response Protocol (Version 1).

Response protocol (version 1) included a 3-tiered stratification for stress scores (ratings: 0–4, 5–7, 8–10). During PDSA cycle testing of the response protocol, it became clear that the 3-tiered stratification added increased complexity without helping guide next steps in care (Fig. 3).

#### Response Protocol (Version 2).

Response protocol (version 2) removed the 3-tier stratification and established a cutoff score of ≥5 that activated a family stress response protocol. The protocol included the following: (1) nursing/physician huddle (in-person, by phone, or via email); (2) expedited referrals for hospital and/or community support services; and (3) personalization based on each family’s unique needs by asking the question, “*What would be most helpful for you and your family right now?*”

During the screening process, staff identified psychosocial safety concerns related to postpartum depression, suicidal ideation, or safety issues in the home. As a result, we created an escalation protocol for psychosocial safety risk whereby staff was trained to escalate concerns to unit nursing and physician leadership and involve child psychiatry and/or hospital security when needed.

### Team Coordination, Communication, and Documentation

Early PDSA cycles were conducted on paper (Fig. 3). To improve efficiency and streamline documentation, we recorded family stress scores and support interventions on a standardized progress note template in the EHR. Furthermore, a Health Insurance Portability and Accountability Act–compliant email distribution list was used to expedite referrals for psychosocial services and to facilitate team communication and coordination. The email distribution list included intensivist and nursing leadership, nursing champions, social work, child life/CAT, integrative health, spiritual care, child psychology, and psychiatry providers. Greater levels of screening and response reliability were achieved through the creation of a FST flowsheet in the EHR, which we then integrated within social work, nursing, child life/CAT, integrative health, and chaplaincy departments’ standard initial assessments for hospitalized patients.

### Youth and Family Engagement to Improve Communication and Interdisciplinary Staff Education

Early in the improvement work, PICU staff identified that a major barrier to asking families about stress was that they felt they would be “opening Pandora’s Box” without having the ability to mitigate or address families’ concerns. To address this anticipated barrier, family advisors worked collaboratively with PICU staff to create an FST frequently asked questions sheet (Fig. 3), which provided scripted language for staff to consider when introducing the screening tool to families. Family advisors shared that the simple act of asking families about their stress and addressing their concerns would likely decrease stress and encourage ongoing dialogue between the family and clinical team. Feedback incorporated from family advisors from diverse backgrounds helped increase the cultural acceptability and relevance of the screening tool. The FST has been translated into our hospital’s top 10 languages and is conducted with live or video interpretation when needed.

### Process Measures

**Fig. 4.**
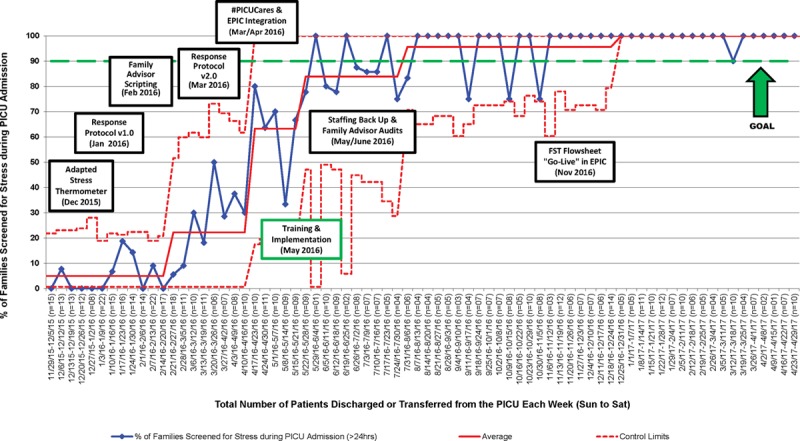
Process control P-chart of the % of families screened for stress during PICU admission. EPIC; electronic health record.

The percentage of families screened for stress increased from 0% to 100% over 18 months (Fig. 4). The percentage of families with stress scores ≥5, who received recommended interventions based on the family stress response protocol, increased from a baseline rate of 0% to 100% during the 18-month QI initiative. Based on a chart review from June to July of 2016 after unit-wide FST implementation, 100% of families with stress ratings ≥5 received psychosocial support services from social work (85%), integrative health (65%), child life/CAT (53%), and chaplaincy (44%), within 24 hours of an elevated stress rating. We identified safety concerns requiring escalation to unit leadership, child psychiatry, and hospital security in 6% of families with elevated stress scores.

### Outcome Measures

Parent-reported family satisfaction scores for “degree to which staff addressed your emotional needs” increased from a mean score of 81.7 in 2015 to 87.3 in 2017 (*P* < 0.01; 95% CI). We extracted satisfaction scores from the Press Ganey database. From 2015 to 2017, the number of security calls for distressed families and visitors of pediatric patients decreased by 50% from 10 calls in 2015 to 5 calls in 2017.

## DISCUSSION

### Summary

The successful implementation of a co-designed family stress screening tool and response protocol led to the timely coordination of parent support interventions, the improved family perception of emotional support, and reduced family crises in the PICU. We speculate that the success of our intervention implementation was enhanced by the engagement of family advisors and interdisciplinary team members.

### Interpretation

The most impactful interventions in helping us achieve our aims were the EHR integration of the FST flowsheet within standard initial assessments for social work, child life/CAT, and nursing, and the identification of the pediatric social worker as the designated family stress screener with child life and nursing staff providing task assistance when needed. When compared with prior publications on parental stress in the PICU, our findings address a critical gap in the literature with the implementation of universal screening for family stress, early identification of psychosocial risks, and timely coordination of support interventions within the PICU context. Another unique contribution to the literature is the integration of the family advisors role within the QI team and co-design approach.

Contextual elements associated with improvement success included leadership support for the family experience, quality, and resilience initiatives; technology infrastructure for EHR integration and data reporting; interdisciplinary team and family advisor engagement; and nursing champion involvement. An unintended consequence was the use of the FST EHR flowsheet by staff working in other units, where the FST screening tool had yet to be formally introduced, facilitating early spread across other pediatric units. The team was unable to implement a consistent family stress reassessment protocol despite multiple PDSA cycles.

The project resulted in newly created FST-related roles and the responsibilities, which are now shared across the interdisciplinary team. Hospital investments in building more robust, integrated psychosocial support services allowed for greater capacity and collaboration across disciplines. The percentage of families screened for stress in the PICU has been sustained at 100% for over 2 years. Over the past year, the FST screening and response system have been adapted and spread to the congenital cardiovascular care unit, neonatal intensive care unit, pediatric acute care unit, pediatric inpatient rehabilitation, and ambulatory chronic illness care.

### Limitations

Our QI initiative had several limitations. The availability of social work staff to collect family stress ratings and facilitate support interventions was critical for success; therefore, other institutions may not be able to incorporate a similar process. Staff members personalized their introduction to the FST and approach to gathering information, which may have led to some variation in the use of the stress scale and measurement bias. Families with PICU admissions under 24 hours were not included in the new process and may have unique needs and stressors. Finally, given the lack of a consistent reassessment protocol, we were unable to assess which family support interventions were most effective in decreasing family stress or how stress scores correlated with a child’s medical condition and care.

## CONCLUSIONS

To our knowledge, this improvement initiative is the first to implement and sustain the use of a co-designed family stress screening tool and response protocol in the PICU. Given the potential wide-ranging, long-term psychologic effects of family distress on both the child and their family following PICU discharge, it is critical that we create universal systems for the early identification of family distress and effective interventions to enhance family coping and resilience. The ability to use a simple family stress screening tool across the full continuum of care has significant implications for practice and in deepening our understanding of family stress, coping, and resilience in the context of childhood illness.

## ACKNOWLEDGMENTS

The authors thank our Sala Institute family partners (Beth Silber, Erik Ward, Family Advisory Council, and Youth Advisory Council), Hassenfeld Children’s Hospital at NYU Langone and Sala Institute Leadership (Michele Lloyd, Fiona Levy, Juliette Schlucter, Lucy Pereira-Argenziano, Liza Cooper, Arun Chopra, and Linda Zieman), Pediatric Intensive Care Unit Nursing Staff (Tiffany Folks, Mary Rose, Guerline Dalrymple, Theresa Yarri, Lauren Selikoff, Christy Perkins, Caitlin Coit, Kelsey Hanrahan, Lauren Arrigoni, and Mary Ellen Sheldon), Department of Pediatrics (Rebecca Rosenberg), Department of Social Work (Dara Weiss, Lindsey Drewry, Deborah Dore, Erin Villani, and Erin Lauinger), Department of Therapeutic Recreation, Child Life & Creative Arts Therapies (Ingrid Olsen-Gallagher, Jami Barretta, Megan Walsh, and Stacey Schneider), Integrative Health Services (Amy Eberhardt, Jeanne Abatemarco, and Taryn DeSio), Chaplaincy Services (Matthew Dimick), and the Department of Child and Adolescent Psychology & Psychiatry (Yamalis Diaz, Rebecca Lois, and Aron Janssen) for their contributions to the project and assistance with the study.

## DISCLOSURE

The authors have no financial interest to declare in relation to the content of this article.
